# The Prospects for a Monist Theory of Non-causal Explanation in Science and Mathematics

**DOI:** 10.1007/s10670-020-00273-w

**Published:** 2020-05-30

**Authors:** Alexander Reutlinger, Mark Colyvan, Karolina Krzyżanowska

**Affiliations:** 1 Munich Center for Mathematical Philosophy, Ludwig-Maximilians Universität, Ludwigstr. 31, 80539 Munich, Germany; 2 University of Sydney, A14 Main Quadrangle, Sydney, NSW 2006 Australia; 3 Institute for Logic, Language and Computation, University of Amsterdam, Science Park 107, 1098 XG Amsterdam, The Netherlands; 4 Arché Research Centre, University of St Andrews, St Andrews, Scotland, UK

## Abstract

We explore the prospects of a monist account of explanation for both non-causal explanations in science and pure mathematics. Our starting point is the counterfactual theory of explanation (CTE) for explanations in science, as advocated in the recent literature on explanation. We argue that, despite the obvious differences between mathematical and scientific explanation, the CTE can be extended to cover both non-causal explanations in science and mathematical explanations. In particular, a successful application of the CTE to mathematical explanations requires us to rely on counterpossibles. We conclude that the CTE is a promising candidate for a monist account of explanation in both science and mathematics.

## Lange’s Challenge

Since the late 1980s the attention of philosophers interested in theories of explanation has been almost entirely on causal explanations and causal theories of scientific explanation. However, the tide has turned: many philosophers of science and philosophers of mathematics in the current debate on explanation agree with the view that both causal and non-causal explanations exist (see Reutlinger 2017a; Mancosu 2018 for overviews of the current literature). This recent debate, however, has focussed almost exclusively on philosophical accounts of non-causal explanations *in the sciences*.^1^

One prominent account of non-causal (and causal) explanations in the sciences consists in (different versions of) the counterfactual theory of explanation (CTE henceforth). The key idea of the CTE is that causal as well as non-causal explanations provide information about how the explanandum counterfactually depends on the explanans. We believe that this development constitutes progress in the debate on scientific explanation. (We will provide a detailed exposition of the CTE below.) But even if the CTE is an adequate theory of scientific explanations, the question arises whether it can be expanded to cover explanations in pure mathematics (aka *intra-mathematical explanations*).

The question of extending the CTE to explanations in mathematics is usually overlooked by proponents of the CTE. Indeed, as Lange (2016: 231) correctly points out, non-causal explanations in pure mathematics have not received sufficient attention and there is very little by way of any philosophical accounts of explanation in mathematics. Clearly, the sciences are not the only intellectual projects striving for explanation, be it causal or non-causal. Pure mathematics is also in the business of providing explanations—that is, explanations of why a particular mathematical statement is true. And, indeed, Lange’s current work (e.g. Lange 2014, 2016, 2018) makes significant steps towards an analysis of mathematical explanations—thus building on work by Steiner (1978a, b), Kitcher (1981) and Colyvan (2012).^2^

Lange suggests that the CTE cannot capture non-causal explanations in pure mathematics (2016: 87–88, 307; 2018: Sect. 2).^3^ What is the source of the problem of applying the CTE to explanations in pure mathematics? According to Lange, the problem rests on the kind of necessity attached to the explanatory assumptions in mathematical explanation: “they possess an especially strong variety of necessity and therefore have an especially strong resistance to being changed” (Lange 2016: 88). Indeed, Lange’s objection seems to be natural and prima facie compelling because, after all, mathematics is usually taken to be a body of necessary truths. What kind of necessity could have a “stronger resistance to being changed” than the necessity of pure mathematics? One way to articulate this objection relies on specific semantics for counterfactuals (although Lange does not explicitly do so): a counterfactual whose antecedent expresses an impossible proposition (such as ‘if mathematical statement *p* were false’) is trivially true (for instance, Lewis 1973; Stalnaker 1968). Let us rephrase Lange’s objection as a challenge to the CTE:

**Lange’s Challenge:** Proponents of the CTE must show that their theory of explanation is applicable to explanations in pure mathematics.

This is a serious challenge and we are convinced that anyone defending the CTE as a general account of explanation has to respond to it.^4^ Taking the CTE’s success regarding scientific explanations for granted, we will defend the claim that a broadly CTE-approach to explanation is able to capture explanations in pure mathematics. The approach we explore is one utilising *counterpossible conditionals*.

We will proceed as follows: in Sect. 2, we will provide a definition of monism and suggest that the CTE is currently one the most promising monist approaches to explanation. In Sect. 3, we reconstruct the CTE in a way that is supposed to remain neutral with respect to different versions of it. Supposing that the CTE has been successfully applied to causal and non-causal explanations in science, we argue that our ‘neutral’ reconstruction of the CTE is also applicable to two examples of mathematical explanation. Section 4 deals with a consequence of Sect. 3: if one expands the CTE to mathematical explanations, one has got to provide a semantics for counterpossibles. We simply point out that advocates of the CTE can rely on already existing work on a non-standard semantics for counterpossibles and we provide a sketch of one version of such a semantics. Section 5 presents an independent argument for extending the CTE to mathematical explanations. It is not merely a requirement of the CTE to use counterpossibles, at least in the context of mathematical explanation. We provide evidence for the claim that mathematicians also do in fact appeal to counterpossibles in their reasoning. This evidence lends additional support to our proposal of extending the CTE from scientific to mathematical explanations. In Sect. 6, we sum up what has been achieved with respect to a defence of a CTE-based monism.

## Monism Versus Pluralism

Why is it important to address Lange’s challenge? The challenge brings into focus an important question about one of the big-picture issues in the present debate on explanation in science and pure mathematics: the issue of whether one should be a *monist* or a *pluralist* about explanation. If there are causal and non-causal explanations in the sciences and non-causal explanations in pure mathematics, what does it mean to be a monist or a pluralist with respect to explanation? We follow Reutlinger’s (2017a, b) and Reutlinger and Saatsi’s (2018a, b) exposition of the distinction between monism and pluralism and we think it is helpful to take Lange’s work as a starting point to illustrate this distinction.^5^

Lange favours a kind of explanatory pluralism. As we understand Lange’s project, his discussion of a variety of case studies of scientific and mathematical non-causal explanations mainly serves the purpose of gathering evidence for explanatory pluralism. Lange describes his pluralist approach in various places. For instance:

I will not try to portray non-causal scientific explanations as working in roughly in the same way as causal scientific explanations do (except that some variety of non-causal dependence appears in place of causal dependence). I will not even try to portray all non-causal scientific explanations as working in the same way as one another. (Lange 2016: xii)

Summarising the results of his book, Lange writes:

I have not argued that every example of explanation in math or every example of non-causal scientific explanation falls into one of the kinds of non-causal explanations I have identified. I have also not tried to force all of the explanations into a single narrow mould. (Indeed I see no good reason to award any greater degree of plausibility to a proposed ‘model’ of explanation in math and science [...] just because it purports to offer the same account of all examples.) However, I have tried to group the examples that I have studied into various kinds based on how the explanations work, and I have also tried to highlight some of the affinities among these kinds of explanation. (Lange 2016: 371)

It is appropriate to characterise Lange’s explanatory pluralism and explanatory pluralism in general as follows: a pluralist holds that, first, there are different types of explanations (in this particular debate, causal and non-causal explanations) in the sciences and in pure mathematics, and that, second, there is no single theory of explanation covering all of these types of causal and non-causal explanations; instead one needs two (or more) distinct theories of explanation to adequately capture all causal and non-causal explanations.

Monists agree with pluralists that there are (prima facie, at least) different types of explanation—here, causal and non-causal ones. However, monists claim that—contrary to pluralists—there is indeed one single philosophical account capturing both causal and non-causal explanations in the sciences and in pure mathematics. Monists hold that causal and non-causal explanations share at least one feature that makes them explanatory.

Perhaps the most promising and most elaborate recent attempt to make progress on a monist approach to explanation comes from counterfactual theories of causal and non-causal explanations. Proponents of the counterfactual theory have articulated and explored this approach in application to various examples of non-causal explanations in science (Bokulich 2008; Kistler 2013; Saatsi and Pexton 2013; Pexton 2014; Pincock 2015; Rice 2015; Reutlinger 2016a, b, 2018; Saatsi 2018; French and Saatsi 2018; Woodward 2003, 2018).

There has also been some preliminary work of applying the CTE to mathematical and logical explanations (Baron et al. 2020). Traditionally, Hempel’s covering law account and Kitcher’s unification account were candidates for monist accounts of explanation but both of these accounts face notorious problems in the context of (causal) scientific explanations (see Woodward 2017; Salmon 1989).^6^

As a referee correctly pointed out to us, monists propose necessary and sufficient conditions for explanations that are satisfied in the case of both causal and non-causal explanations. But monists might still want to draw a distinction between causal and non-causal explanations. In this paper, it is not our goal to argue in favour of one particular strategy for drawing such a distinction. But consider two examples of how this might be accomplished. First, Hempel’s covering-law account is an instructive historical example for illustrating monism (Hempel 1965: 352). Hempel argues that causal and non-causal explanations are explanatory by virtue of having (at least) one feature in common: nomic expectability. In the case of causal explanations, one expects the explanandum to occur on the basis of causal covering laws (laws of succession) and initial conditions; in the non-causal case, one’s expectations are based on non-causal covering laws (laws of coexistence) and initial conditions. Second, if one accepts a counterfactual theory of explanation, non-causal explanations are explanatory by virtue of exhibiting non-causal counterfactual dependencies; causal explanations are explanatory by virtue of exhibiting causal counterfactual dependencies. Proponents of the counterfactual theory propose different strategies for drawing a distinction between causal and non-causal counterfactual dependencies (for a comprehensive overview, see Reutlinger 2017a: section 3.3).

Why should one prefer monism to pluralism? We believe the answer is straightforward: prima facie, monism is superior to pluralism, if one assumes that, ceteris paribus, philosophers prefer more general philosophical theories to less general theories.^7^ Monism promises one general theory of causal and non-causal explanations in science and mathematics, while the pluralist alternative is piecemeal, offering different, less general accounts of explanation in various cases. For this reason, we take it that monism is an attractive view deserving further exploration. However, recalling Lange’s challenge, our central question is whether monists are able to deliver a plausible account of explanations in pure mathematics.

## Extending the CTE to Mathematical Explanations

As already indicated, we hold that arguably the most promising monist approach is the counterfactual theory of explanation (CTE). Current counterfactual theories typically take Woodward’s counterfactual account of causal explanations as their starting point:

An explanation ought to be such that it enables us to see what sort of difference it would have made for the explanandum if the factors cited in the explanans had been different in various possible ways. (Woodward 2003: 11)

Woodward’s version of the counterfactual theory of explanation and its underlying interventionist theory of causation is originally intended to capture causal explanations (Woodward 2003: 203). However, the core idea of the counterfactual theory—that is, analysing explanatory relevance in terms of counterfactual dependence—is not necessarily tied to a causal interpretation. Indeed, Woodward suggests this line of argument, although without pursuing the idea any further:

[T]he common element in many forms of explanation, both causal and non-causal, is that they must answer what-if-things-had-been-different questions. (Woodward 2003: 221).

To answer what-if-things-had-been-different questions is to reveal how the explanandum counterfactually depends on possible changes in the conditions described by the explanans. Hence, the monist proposal of the CTE is that causal and non-causal explanations are explanatory by virtue of exhibiting how the explanandum counterfactually depends on the explanans (for Woodward’s own recent efforts to develop a CTE-style monism, see Woodward 2018).

In this paper, we will focus on two necessary conditions that different versions of the CTE impose on scientific explanations:

1. **Inference Condition:** The explanans statements allow us to either deductively infer the explanandum statement, or to infer a conditional probability of the explanandum statement given the explanans statements, such that $P(explanandum | explanans)>P(explanandum)$.^8^
2. **Dependency Condition:** The explanandum counterfactually depends on certain possible changes in the conditions described by the explanans (i.e. if the explanans conditions were different, then the explanandum would be different as well).

We focus on these two conditions because they are the common denominator of different versions of the CTE (Bokulich 2008; Kistler 2013; Saatsi and Pexton 2013; Pexton 2014; Pincock 2015; Rice 2015; Reutlinger 2016a, b, 2018; Colyvan 2018; Saatsi 2018; Baron et al. 2020; French and Saatsi 2018; Woodward 2003, 2018; Jansson and Saatsi 2019). We adopt Reutlinger’s useful labels for and reconstructions of these two conditions (2016a: 737, 2016b: 244, 2018: 78–79).

For the purposes of this paper, we will take it as a premise that the CTE applies to non-causal and causal explanations *in science*. That is, we will assume that the CTE is a successful monist account of scientific explanation. In this paper, it is not our goal to defend the CTE as an account of (non-causal and causal) *scientific* explanation. This work has been done elsewhere—for instance with respect to symmetry explanations and renormalisation group explanations (see references in the previous paragraph above). In Sect. 3, we will address our main question whether the success of the CTE can be extended from non-causal explanations in science to non-causal explanations in *pure mathematics*.^9^ We will discuss this question in light of two examples of explanations in mathematics in Sects. 3.1 and 3.2.

### Explaining the Intermediate-Value Theorem

Consider an example of a mathematical explanation of why the intermediate-value theorem holds. Recall that the intermediate-value theorem states that if *f* is a real-valued function continuous on a closed interval [*a*, *b*] and *c* is any number between *f*(*a*) and *f*(*b*) (inclusive), then there exists a *z* in [*a*, *b*] such that $f(z) = c$. The intermediate-value theorem holds because the image of the interval [*a*, *b*] under *f* is connected (since the image of a connected set under a continuous function is also connected) and *c* is in this connected set, since it lies between *f*(*a*) and *f*(*b*) (Apostol 1967). Connectedness (of the image of interval [*a*, *b*] under *f*) is what is doing the immediate explanatory work but it turns out that continuity is the key, since it is continuity that guarantees the connectedness of the image in question. After all, from what is given in the conditions of the theorem we have no other reason to expect that the image of [*a*, *b*] under *f* is connected; it is the continuity of *f* that ensures the connectedness of the set of interest.

We can reconstruct this explanation as having the familiar explanans-explanandum structure: what we wish to explain here (the explanandum) is the theorem itself (or why it holds). The explanans appeals to the notions of *connectedness*, and *continuity*. That is, these notions constitute the core part of the explanans. Consider continuity and the crucial role it plays. The definition of a continuous function at play here is as follows: a function $f: X \rightarrow Y$ is continuous iff the pre-image of every open set in *Y* is open in *X*. This is the *topological* definition but there are others (e.g. the well-known $\epsilon$–$\delta$ definition, which is a special case of the topological definition). Any of these usual mathematical definitions can be used to support the proof of the intermediate-value theorem, because such definitions guarantee the connectedness of the image of [*a*, *b*] under *f*.

But there are also other possible definitions of continuity that have not been taken up in standard mathematics.^10^ For example, suppose that space-time is discrete, then standard $\epsilon$–$\delta$ notions of continuity would not serve us well. We could either stick with our standard definitions and hold that there is no continuous motion or we could adopt a different notion of continuity. We contend that it would be very reasonable to follow the latter path and that such definitions of “continuity” would not support a proof of the intermediate-value theorem.

Now, what would it take for this mathematical explanation to satisfy the conditions of the CTE? The CTE applies to the presented explanation of why the intermediate-value theorem holds if the following statements are true:

1. The *Inference Condition* is met, because the explanans statements (mainly consisting of appeal to the standard notions of *connectedness*, and *continuity*) deductively entail the explanandum (the intermediate-value theorem).
2. For the *Dependency Condition* to be satisfied the following counterfactual has to be true:
  (CF1) “If continuity were defined in a non-standard way, then the intermediate-value theorem would not hold.”

In the context of this explanation, we hold that the Inference Condition is satisfied, because the explanation has the form of deductively valid proof.

The question, however, is whether the Dependency Condition is met in the case of the example. What the explanation sketched above indicates is that we can entertain counterfactuals associated with the explanation of the intermediate-value theorem; counterfactuals that involve apparently impossible statements as: “suppose that *g* is a continuous function that violates the intermediate-value theorem” (such as CF1 above). We could entertain such a function by appeal to an impossible situation or by appeal to a different definition of continuity (perhaps, motivated by contingent features of the structure of space-time).^11^ Either way, we have counterfactuals/counterpossibles associated with our explanation of the intermediate-value theorem, at least if one favours the CTE as an account of explanation.^12^ We will turn to the issue of such conditionals in Sect. 4.

### Explaining Why You Can’t Square a Circle

Consider a second example of a mathematical explanation: the explanation for the impossibility of squaring the circle. According to the CTE, this explanation involves appeal to counterpossibles.

It is well known that it is impossible to construct a square of the same area as given circle, using only a straight edge and compass.^13^ The reason is surprising: $\pi$ is a transcendental number. That is, $\pi$ is not the root of any polynomial with (non-zero) rational coefficients. A sketch of the proof will help us see how the transcendentalness of $\pi$ explains this famous impossibility result. Since the area of a circle is $\pi r^2$, where *r* is the radius of the circle, constructing a square of the same area amounts to constructing a square with sides $\sqrt{\pi }r$. Now consider the constructions one can make with straight edge and compass. It turns out that only the following constructions can be made (where *a* and *b* are lengths constructed via straight edge and compass): $a + b$, $a-b$, *ab*, *a*/*b*, and $\sqrt{a}$. We now switch to abstract algebra to look at the algebraic structure of these geometric constructions. We can show that the constructible lengths form a field. It follows that all rational lengths can be constructed and any constructible length is algebraic—that is, it is the root of some polynomial with (non-zero) rational coefficients. The 1882 proof by Lindemann that $\pi$ is transcendental completes the proof that the circle cannot be squared (Bold 1982).

We can reconstruct this explanation as having the following explanans-explanandum structure: the explanandum statement is that it is impossible to construct a square of the same area as a given circle, using only a straight edge and compass. The explanans consists of the following key mathematical statements: (a) the area of a circle is $\pi r^2$, (b) constructing a square of the same area amounts to constructing a square with sides $\sqrt{\pi }r$, (c) all rational lengths can be constructed and any constructible length is algebraic (that is, not transcendental), and (d) $\pi$ is transcendental. Statements (a) and (b) amount to definitions of the area of the geometric figures relevant in the context of the explanation (circle and square).

Again, what would it take for this mathematical explanation to satisfy the conditions of the CTE? The CTE applies to the no-squaring-the-circle explanation if the following statements are true:

1. The *Inference Condition* is met, because the explanandum (the impossibility result) is deductively entailed by the explanans statements (a)–(d).
2. For the *Dependency Condition* to be satisfied something like the following counterfactual will be the key:
  (CF2) “If $\pi$ were an algebraic number (and not transcendental), then the circle could be squared.”^14^

We take it that, being a mathematical proof, the explanation of the impossibility of squaring the circle straightforwardly satisfies the Inference Condition. However, as noted, the crux is satisfying the Dependency Condition. We will turn to this topic in the next section and argue that the Dependency Condition can in fact be met in the case of non-causal mathematical explanations, if one relies on counterpossibles and an appropriate semantics for them. If this is right, the prospects for monism about explanation are in good shape.

## Counterpossibles Without Tears

The apparent problem for extending the CTE to the mathematical case is that it is hard to make sense of the counterfactuals in question. More specifically, satisfying the dependency condition looks problematic: one needs to consider what if things had been different. In the mathematical case, this involves supposing that mathematical facts were different. But on the standard philosophical accounts of mathematics, mathematical truths are necessary. So the counterfactuals that are at the core of the CTE would seem to be deeply problematic in the mathematical case. The counterfactuals in question are those such as CF1 and CF2 figuring in the last section. Indeed, another way of stating Lange’s challenge is thus: *the CTE trivialises in the case of mathematics; so it is of no use*. After all, according to the standard semantics for counterfactuals, any counterfactual with an impossible antecedent is trivially true because there is no possible world where the antecedent is true (Lewis 1973 and Stalnaker 1968). Thus, the counterfactual:

(CF3) “Had Fermat’s Last Theorem been false, Munich would not be in Germany.”

would be true. We will argue that the problem we are encountering here is with the standard semantics for counterfactuals not with the CTE.

First we note that we *can* make sense of mathematical counterfactuals. For example:

(CF4) “Had Fermat’s Last Theorem been false, there would be positive integers, *a*, *b*, and *c* and some integer $n>2$ such that $a^n + b^n = c^n$.”

Indeed, this is what it would be for Fermat’s Last Theorem to be false, hence the counterfactual CF4 is true. But other counterfactuals such as CF3 are false. In short, CF4 is true but not trivially true and CF3 is false (not trivially true)—contrary to the standard semantics for counterfactuals. Of course, we now know that Fermat’s Last Theorem is true so the counterfactuals in question may seem slightly odd. But consider an open problem in mathematics such as the existence of quasiperfect numbers.^15^ There are theorems involving the two possible cases here: quasiperfect numbers exist or they do not. In one of these cases the theorems involve counterpossibles. For example, if, in fact, there are no quasiperfect numbers, then a theorem about their existence is based on an impossible assumption. But the corresponding counterfactual:

(CF5) “Were quasiperfect numbers to exist then they would all be greater than $10^{35}$ and have at least 7 distinct prime factors.”

is not trivially true (see Hagis and Cohen 1982).

In a nut shell, the standard semantics for counterfactuals is ill-equipped to deal with impossible antecedents—they were not designed to deal with such cases. What we need is some way to evaluate *counterpossibles*: counterfactuals such as CF1-CF5. Indeed, in light of the discussion above, it might be argued that we need such an account, irrespective of any ambitions of having CTE as our monist theory of explanation.

Thankfully there are a number of ways of extending something like the standard account of counterfactuals to counterpossibles. Indeed, this can be done with very little effort by way of non-classical logical machinery. One needs to allow impossible worlds in addition to possible worlds. But that is about it. Here we sketch one such way.

In the remainder of this section, we will suggest that if proponents of the CTE want to meet Lange’s challenge and are, thereby, committed to counterpossibles, then they can rely on a ‘non-standard’ semantics for counterfactuals and, in particular, counterpossibles. Note that we will not defend such a semantics here, we will rather take it as a premise (see Baron et al. (2020) for further elaboration, further defence of this approach to mathematical counterpossibles, and further references).

Consider a garden-variety counterfactual such as:

(CF6) “Had the plate not been dropped, it would not have broken.”

The core moves for assessing the truth of a counterfactual such as CF6 amount to three steps: (i) hold some class of facts fixed, (ii) vary (or “twiddle”) some other facts in order to make the antecedent of the counterfactual true, then (iii) consider the downstream consequences of the varying facts for the facts not held fixed.

With a counterfactual such as CF6, it is clear how these three steps apply. We typically hold the past history of the universe fixed, along with the relevant laws. It is worth noting that we need to make *a choice* as to what we hold fixed—for instance, how much of the past history of the universe we hold fixed needs to be decided. The twiddle also involves choices. Obviously we need to vary events from the actual world in order to make the relevant antecedent true (i.e. the plate not being dropped) and there are many ways to do this. For the most part, the variety of ways a plate can fail to be dropped do not matter but this overdetermination can lead to problems.^16^ With good choices of what to hold fixed and what to twiddle, the consequences, should, in most cases, be a matter of inspection (as it were).

Except for the reference to events in the above description, we can use the same three-step procedure in mathematics. In particular, we can assess counterpossibles such as, say, CF1, CF2, and CF3 by: (a) holding most of mathematics fixed, (b) varying the transcendentalness of $\pi$, varying the standard definition of continuity, or varying the truth of Fermat’s Last Theorem and (c) see what follows from (a) and (b). Of course, the twiddle at step 2 involves an impossibility (at least on standard philosophical accounts of mathematics) but it turns out that this fact does not change things very much at all. Given that it is true that $\pi$ is transcendental, the standard definition of continuity holds, and that Fermat’s Last Theorem is false, how do we bring it about that these mathematical statements are false? We do not need to; we only need to *suppose* that they are false. We no more need to *make it the case* that $\pi$ is not transcendental, that continuity is defined in a non-standard way and that Fermat’s Last Theorem is false than we need to make it the case that a dropped and broken plate was not dropped.

What of assessing the downstream consequences for the non-fixed facts after the twiddle? Since the twiddle results in an impossibility, one might think that this means that all hell breaks loose and there is no sensible way of getting non-trivial consequences from the impossible antecedent. This is simply not so. Trivialism follows for the standard account of counterfactuals and, indeed, many logical systems are ‘explosive’—that is, any arbitrary proposition follows from a contradiction in such logics (e.g. classical logic and intuitionistic logics). In paraconsistent logics, however, this is not the case. If needed, we can appeal to paraconsistent logics and impossible worlds.^17^ But it is not clear that anything of the sort is needed to get the basic idea. We can see that were $\pi$ not transcendental (or were Fermat’s Last Theorem false), it would not follow that Munich would be anywhere other than in Germany. We can appeal to a paraconsistent logic to bolster such claims but there really is no need. This is just as obvious as the inference about the plate not breaking had it not been dropped.^18^ The point is that, just as with regular counterfactuals, we typically do not need to follow the ramifications of the twiddle through to its logical closure (or in causal cases, follow its causal history back to the big bang). We simply stipulate that the antecedent is true and look at (only) the relevant downstream consequences.

There are other concerns you might have about this proposal for assessing the truth of counterpossibles but it would take us too far afield to fully defend the proposal here. And besides, such a defence has already been provided (Baron et al. 2020).

What have we achieved? We have argued that proponents of the CTE who care to meet Lange’s challenge can rely on proposals for generalising the standard semantics for counterfactuals to cover counterpossibles in a non-trivial way. If one is convinced by such a semantics of counterpossibles, then the—seemingly problematic—Dependency Condition of the CTE can be satisfied *even* in the mathematical case. If so, this is a big step towards defending the CTE as a monist account of explanations in science and mathematics.

In the next section, we go beyond the requirements that the CTE imposes on explanations: we will examine whether there is evidence that mathematical practice involves appeal to counterpossibles.

## Do We Need Conditionals in Mathematics?

In Sects. 3 and 4, we argued that, according to the CTE, mathematical explanations involve counterpossibles. Our argument might give rise to an interesting objection: the CTE diverges from actual mathematical theorising, because mathematicians do not use—or do not need to use—anything quite so exotic as counterfactuals and counterpossibles. Perhaps all they are doing is looking at deductive inferences from various assumptions. Some of the assumptions turn out to be true and some turn out to be false. According to this objection, there is no need for putting counterpossibles or, indeed, any conditionals in the mouths of mathematicians.^19^

This objection includes two related worries that should be distinguished here. The first is a claim about mathematical practice: mathematicians do not, in fact, use such counterpossible language. The second is the modal claim that there is no need for mathematicians to use such counterfactuals. As we will show in this section, mathematicians do seem happy to use conditionals, including counterfactuals and counterpossibles. This, of course, does not show that they are right in using such language. There may be some reconstruction of their practice along the lines of the suggested objection, appealing only to assumptions and deductive consequences without appeal to conditionals. Be that as it may, the fact that mathematicians do use counterfactuals in such circumstances (as we will show) gives us prima facie reason to take such counterfactuals seriously and we are reluctant to engage in too much reconstruction or reinterpretation of mathematical practice—at least not without good reason.

To avoid misunderstandings, it is not the purpose of this section to provide more examples of explanation in mathematics. What we are going to demonstrate instead is something more general, namely, that mathematicians do use counterfactual conditionals, and counterpossibles in particular, in their writings, and that their choices regarding the grammatical form of their statements do not seem to be accidental. In other words, we want to show that counterfactual and counterpossible conditionals are not just idiosyncrasies of philosophical reconstructions (such as the CTE).

One way to empirically determine if a certain linguistic community speaks in a particular way is to search the corpus of their language, that is, a collection of texts (typically records of both written and spoken word) produced by that community. Since the goal of finding out if mathematicians use counterfactual language is relatively modest, our pilot corpus study did not need to employ any sophisticated, computational methods developed in the field of corpus linguistics. Instead, we searched through a sample of texts written by mathematicians to find examples of the kind of language use we are interested in.

The first step in our pilot study was to assemble a corpus consisting of 20 mathematical texts published between 1984 and 2018. We collected three different types of texts:

1. A selection of research papers exploring, among other things, the consequences of as yet unproven hypotheses, such as Riemann hypothesis, or of assuming the existence of impossible mathematical objects, such as the field with one element.
2. Essays collected in an anthology of “survey papers presenting the status of some essential open problems in pure and applied mathematics, including old and new results as well as methods and techniques used toward their solution.” (Nash and Rassias 2016, p. v.)
3. Undergraduate and graduate textbooks and lecture notes introducing students to various fields of mathematics, such as: Analysis, Calculus, Geometry, or Number Theory.

The selection of the texts was dictated partly by their availability in digital form via open access resources such as arXiv.org, researchers’ personal webpages, or resources accessible through the university libraries’ subscriptions, such as, SpringerLink. In these texts, we searched for the occurrences of subjunctive conditionals. Since the purpose of the study is to argue against the claim that counterfactual language plays no role in mathematical practice, a single instance, in principle, makes the point. For this reason, we will only present a number of examples showing that mathematicians do not only use conditional and counterfactual language, but also, that they use it purposefully, leaving any quantitative analyses for future studies.

To facilitate the search, we focused on the paradigmatic surface structure of a subjunctive conditional, that is, we looked for the sentences consisting of an *if*-clause and a main clause involving the auxiliary ‘would.’ We ended up with a list of 42 conditionals of the form ‘if it had been the case / if it were the case that $\varphi$, then it would be / it would have been the case that $\psi$.’ More specifically, we found instances of counterfactual language in 11 out of 17 essays collected in Nash and Rassias (2016), summing up to total 21 conditionals, in 4 out of 12 research papers, total 7 conditionals, and in 6 out of 7 textbooks, total 14 conditionals. It is then an empirical fact that mathematicians use counterfactual conditionals in their writing. In fact, mathematicians use conditionals both in indicative and subjunctive moods, depending on what they are writing about.

For instance, in a paper on the consequences of the Generalised Riemann Hypothesis by Deshouillers et al. (1997), we can find the following statements:

1. “If the Generalized Riemann Hypothesis holds, then every odd number above 5 is a sum of three prime numbers.” (p. 99)
2. “If the primes up to $10^8$ were uniformly distributed, which they are not, a proportion of about $0.885^2$ of the even numbers would not be covered by [the set of even numbers] $\mathcal {F}_2$.” (p. 102)

The paper is devoted to “The 3-Primes Problem,” that is, the question whether every odd number greater than 5 can be written as a sum of three prime numbers. The sentence (1) is the main theorem of the paper, while (2) occurs in the context of a presentation of a computer search method for verification of the Goldbach conjecture on a given interval [*a*, *b*] (which in the authors’ own experiments was an interval of the length of $10^8$), involved in the proof of (1). Since the Riemann Hypothesis has not been proven one way or another, the truth value of the antecedent of (1) is unknown, hence the use of the indicative conditional is a natural choice.^20^ By contrast, when the authors entertain an antecedent which is not only false, but also known to be false, such as “the primes up to $10^8$ are uniformly distributed” in (2), they choose to phrase the dependency between this assumption and whatever follows from it as the subjunctive conditional. Note that (2) is not only a counterfactual but also a counterpossible.

A subjunctive form can also be used when the antecedent is not known to be false, that is, when its truth value is itself an open question, though the choice of subjunctive tends to reveal the author’s belief in its falsehood. Many instances of subjunctive conditionals can be found when mathematicians explore the consequences of not-yet-proven conjectures such as the Riemann Hypothesis mentioned above, The Chromatic Number of the Plane Problem, or, to consider an example more familiar to philosophers, $\textsf {P} \ne \textsf {NP}$.^21^ For instance, in his essay on the $\textsf {P} \ne \textsf {NP}$ hypothesis, Scott Aaronson writes:

(3) “If just one of these problems [i.e. problems that have been shown to be in $\textsf {P}$] had turned out to be both $\textsf {NP}$-complete and in $\textsf {P}$, that would have immediately implied $\textsf {P} = \textsf {NP}$.” (Aaronson 2016: 25)

The counterfactual conditional (3) occurs in a context of an empirical argument for the inequality of two classes of computational complexity, $\textsf {P}$ and $\textsf {NP}$.^22^ This argument rests on an observation that while thousands of problems have been shown to be either in $\textsf {P}$ or to be $\textsf {NP}$-complete, there is not a single one that has been shown to be both. The antecedent of (3) has not been proven to be false—in principle, it might still happen that an $\textsf {NP}$-complete problem will turn out to be in $\textsf {P}$. Yet the use of the subjunctive is appropriate as it corresponds to a belief that is empirically justified and widely shared in the computer science community.

Counterfactual language can also be found in texts that are primarily of a didactic nature, such as undergraduate textbooks to mathematics or lecture notes. Authors use conditionals to explain basic notions, e.g., the notion of logical equivalence:

(4) “If *X* and *Y* are logically equivalent, and *X* is false, then *Y* has to be false also (because if *Y* were true, then *X* would also have to be true).” (Tao 2016a: 311)

More interestingly, conditionals can be used to explain consequences of certain assumptions such as, for instance, the infamous axiom of Universal Specification, that is, an assumption that every property corresponds to a set. Let us define *P*(*x*) as the following property: “*x* is a set and $x \notin x$,” and the set $\Omega$ as a set of all such *x* of which *P*(*x*) is true, that is, a set of all sets that do not contain themselves. In the following passage from a textbook to Analysis, Terrence Tao explains why these assumptions lead to the Russell’s paradox:

(5) “If $\Omega$ did contain itself, then by definition this means that $P(\Omega )$ is true, i.e., $\Omega$ is a set and $\Omega \not \in \Omega$. On the other hand, if $\Omega$ did not contain itself, then $P(\Omega )$ would be true, and hence $\Omega \in \Omega$. Thus in either case we have both $\Omega \in \Omega$ and $\Omega \not \in \Omega$, which is absurd.” (Tao 2016a: 47)

As we emphasised above, it was not the aim of our pilot corpus study to provide more examples of explanations, but to demonstrate that mathematicians use counterfactual language. Nevertheless, textbooks and lecture notes may be considered particularly valuable sources of data on the language used by mathematicians in the context of explanation, given that the primary goal of proofs found in such texts is arguably to explain key ideas to students. In the literature on mathematical education, it has been emphasised that the role of a proof is not restricted to showing *that* a theorem holds, but first and foremost to provide an explanation of *why* a theorem is true (Hanna 1990; Hersh 1993). Unsurprisingly, then, in teaching materials, we can find multiple examples of conditional and counterfactual language used in the context of proofs, particularly the reductio ad absurdum proofs.

For instance, Clark (2002) in his lecture notes on number theory, in the contexts of a discussion of primality tests, presents a proof of a theorem (a converse of Fermat’s Little Theorem) that states that if $m \ge 2$ and for all *a* such that $1 \le a \le m - 1$ it holds that $a^{m - 1}$ is congruent to 1 modulo *m*, then *m* must be prime. The first step of the proof is phrased as an indicative conditional: “if the hypothesis holds, then for all *a* with $1 \le a \le m - 1$, we know that *a* has an inverse modulo *m*, namely, $a^{m-2}$ is an inverse for *m* modulo *m*,” The next step makes use of a theorem proven earlier (p. 72), which is also an indicative conditional, namely: if the product of two integers *a* and *b* is congruent to 1 modulo $m > 0$ then both *a* and *b* are relatively prime to *m*, that is, the greatest common divisor of *a* and *m*, written *gcd*(*a*, *m*), equals 1, and so does *gcd*(*b*, *m*). In virtue of this fact, the first step amounts to an observation that for $1 \le a \le m - 1$, the greatest common divisor of *a* and *m* is 1. Now, to show that *m* must be prime, one can consider the consequences of the assumption that it is not. Such an assumption leads to a contradiction, which is naturally phrased as a subjunctive conditional:

(6) “...if *m* were not prime, then we would have $m = ab$ with $1< a < m$, $1< b < m$. Then $\gcd (a, m) = a > 1$, a contradiction. So *m* must be prime.” (2002: 97)^23^

Again, this conditional is not only a counterfactual, but also a counterpossible: its antecedent is necessarily false.

Although this research does not show that mathematicians *need* to use counterfactuals and counterpossibles, we do have sufficient empirical evidence to support the claim that counterfactual language is used in mathematical writing, including, importantly, didactic texts such as textbooks and lecture notes, which can be said to have, broadly speaking, explanatory goals.^24^ This finding lends additional support to our proposal of extending the CTE from scientific to mathematical explanations.

## Conclusion and Discussion

We have argued for the desirability of a monist account of explanation across scientific and mathematical contexts and for both causal and non-causal explanations. Our candidate monist philosophical account is the CTE. We have not defended this theory as the only, or even the best, candidate for such a monist account. There may be others such as Strevens’ kairetic account (Strevens 2008) or perhaps older theories of explanation (such as the unification or covering law accounts) can be revived for such purposes. We leave these possibilities for others to explore. We have focussed on the CTE for two reasons. First, it is perhaps the current front runner in the literature on scientific explanations so we take ourselves to be adopting a popular account and showing that it can be generalised to accommodate non-causal explanations in both science and mathematics. Second, according to Lange’s challenge, the CTE is thought to have a serious limitation in its ability to accommodate mathematical explanations. We accepted the challenge to show how, with a relatively straightforward move from counterfactuals to counterpossibles (and a semantics for them), the CTE can be applied to mathematical explanation. The latter, in turn, clears the way for the CTE to be a candidate for a monist theory of explanation.

We also note that we have restricted our attention to explanation in science and in mathematics, with our primary attention on the latter. We have not discussed explanations in other areas such as folk discourse, ethics, metaphysics, and logic. Again, there is further work to be done here—perhaps explanatory folk discourse will largely resemble (causal) scientific explanation while the latter three might resemble mathematical explanation in significant ways. In any case, providing an account of mathematical explanation will presumably help in providing an account of explanation in other areas where non-causal and non-contingent matters prevail.^25^

If we have been successful in our arguments thus far, monism about explanation is a live option. Moreover, the CTE looks like a good candidate for such an account. After all, we have argued that the CTE can be applied in both mathematics and science. What we have not done, however, is show that the CTE can handle *all* instances of explanation in science and mathematics. For example, Colyvan et al. (2018) suggest that there might be two quite different kinds of explanation in operation within pure mathematics: one that bears some resemblance to unificationist explanation and one that places emphasis on local relevance. Similarly, it might be argued that no single theory of explanation can work across the board in all scientific contexts.^26^ If so, monism about explanation would be in trouble. It is too early to say much about the kinds of explanations found in mathematics. As we have already noted, there is surprisingly little philosophical work on this topic.

But even so, the arguments of this paper show that monism is not scuttled by the mere fact that there are explanations in mathematics. The CTE can deal with the kind of problems raised by the modality of mathematical explanations. Whether it can deal with all mathematical explanations or, indeed, all scientific explanations is an open question. Be that as it may, in our view, monism remains a live option and one well worth pursing.

## Footnotes

1 However, the current interest in non-causal explanations was nonetheless (partly) initiated by the work of philosophers of mathematics, including, for example, Smart (1990), Colyvan (1998, 2002) and Baker (2005).

2 See Mancosu’s (2018: Sects. 4–7) for an up-to-date overview of the history of debate on explanation in pure mathematics.

3 Lange also argues that the CTE fails to capture at least one important type of non-causal explanation in the sciences which Lange calls “explanation by constraint”. We will set aside the issue of explanations by constraint, since French and Saatsi (2018) and Reutlinger (2018) have already argued how to apply the CTE to such cases.

4 Reutlinger (2016b) already acknowledges this challenge in the context of grounding explanations in metaphysics that are based on grounding facts obtaining with metaphysical necessity. But he does not address the challenge. See also Wilson (2018) for a case for counterpossibles in the service of grounding explanations in metaphysics.

5 Reutlinger (2017a, b, 2018) also present other big-picture views such as causal reductionism and particularism. In this paper, we will focus on the prospects of monism as a competitor to pluralism.

6 Strevens’ (2008) kairetic account is another candidate for a monist account of explanation in the current debate, although we think that more needs to be said to defend the kairetic account as a brand of monism (see Reutlinger [2017a: Sect. 3.3] for a discussion).

7 We will not argue for this methodological assumption here.

8 For our discussion of explanations in mathematics, the probabilistic part of the Inference Condition is not relevant.

9 As one referee pointed out and as Woodward (2003: 220–221) notes, extending the CTE to mathematical explanations can be seen as elaborating a necessary condition that Steiner (1978b: 143) imposes on mathematical explanation: namely, that explanations in mathematics consist in “varying” certain features of a proof, in showing that a theorem “depends” on the assumptions from which it is proven. However, proponents of the CTE do not necessarily have to accept other features and conditions of Steiner’s account (such as the notion of a “characterising property” or the requirement that mathematical explanations have to be “generalisable”).

10 See Colyvan and Easwaran (2008) for discussion of such “physical” notions of continuity.

11 See Lakatos (1976) for discussion of the role of definition in the proofs of mathematical results.

12 Moreover, as a referee pointed out, this example is somewhat different in structure from the others in that here the dependency condition requires us to entertain alternative accounts of continuity rather than have some variable take a different value. We can, however, think of the dependency condition here in terms of a ‘continuity variable’ taking different values, where the values in question are different definitions of continuity. Thought of this way, this example is not so different from the others.

13 As usual, we restrict our attention to Euclidean space here. It turns out that the circle can be squared in certain non-Euclidian spaces such as Gauss–Bolyai–Lobachevsky Space (Gray 1989).

14 Strictly speaking, this is not right and requires suitable qualification; not all algebraic numbers are constructible. It would take us too far afield to specify the extra conditions required on algebraic numbers in order to guarantee that they are constructible. The precise conditions, in fact, do not matter for present purposes but if you prefer, you can replace the counterfactual here with something like: “Were $\pi$ an algebraic number *of the right kind*, the circle could be squared”.

15 The details about quasiperfect numbers do not matter; all that matters is that the existence of such numbers is a genuinely open question. For the record, a *quasiperfect number* is a natural number that is equal to the sum of its non-trivial divisors.

16 Recall examples such as Quine’s “had Verdi and Bizet been compatriots then would Verdi have been French or would Bizet been Italian?” (Quine 1982).

17 In fact, one may not need to drop the classical consequence relation to have a semantics for counterpossibles that does not render them all trivially true—impossible worlds might be all it takes. See Berto et al. (2018) for an example of such proposal.

18 And in the plate case we also rely on intuitions; we do not, nor do we need to, formally deduce—in a specific logic—the non-breaking of the plate or build a detailed physical model to test the claim in question.

19 Hartry Field once suggested (without endorsement) to Mark Colyvan that this might be all that is going on in such cases.

20 However, as we will see, such cases also permit the use of a counterfactual.

21 For more examples of counterfactuals and counterpossibles used in mathematical papers, see, for instance, Aaronson (2016: 3, 4, 25, 27–28, 51); Bennett (2016: 179); Borger (2009: 7, 10); Constantin (2016: 265); Harrison and Pugh (2016: 297); Kauffmann (2016: 305); Manin (2008: 5); Morris and Soltan (2016: 366); Mussardo and LeClair (2018: 9–10, 12); Rosenberg (2016: 386); Saymour (2016: 420, 432–433); Soifer (2016: 454, 470); Szemerédi (2016: 463, 470); Vaughan (2016: 484).

22 Recall that $\textsf {P}$ denotes a class of all decision problems that can be solved by a Turing machine in a polynomial time, which can be said to capture the notion of an “efficient” or “feasible” computation (Arora and Barak 2009: 25–27). $\textsf {NP}$ stands for “nondeterministic polynomial time.” It denotes a class of all decision problems that can be “efficiently verified”, that is, if the answer to a problem is “yes,” then “there is a polynomial-size proof that a Turing machine can *verify* in polynomial time” (Aaronson 2016: 2). While it is clear that the class $\textsf {P}$ is contained in $\textsf {NP}$, since, if a problem can be solved, its solution can also be verified in polynomial time, it remains an open question if this containment is proper or if $\textsf {P} = \textsf {NP}$.

23 For more instances of counterfactuals occurring in teaching materials, see, for instance: Geveci (2016: 70); Guirao et al. (2016: 4, 132); Stein (2009: 14, 82); Tao (2016a: 36, 258); Tao (2016b: 14, 103, 176, 177). Naturally, reductio ad absurdum proofs can be found in research articles, too, e.g. Mussardo and LeClair (2018: 12).

24 A follow-up study could establish whether the counterfactual language is more prevalent in didactic text than in research papers, whose main aim is, typically, to report proofs of new theorems. The results of our pilot study already hint at such a possibility. However, a quantitative study would require a more careful construction of the corpus than the pilot reported here.

25 See Baron and Colyvan (2016) for a discussion of logical explanation and its similarity to mathematical explanation.

26 Recall Lange’s (2016) claim that the CTE cannot be applied to explanations by constraint in science.
